# Factors influencing innovation performance of China’s high-end manufacturing clusters: Dual-perspective from the digital economy and the innovation networks

**DOI:** 10.3389/fpsyg.2022.1012228

**Published:** 2022-09-27

**Authors:** Liping Zhang, Kaiqi Xiong, Xinzhi Gao, Yi Yang

**Affiliations:** School of Economic and Management, East China Jiaotong University, Nanchang, China

**Keywords:** China’s high-end manufacturing cluster, innovation network, digital economy, innovation performance, structural equation modeling

## Abstract

In the era of digital economy, the impact of innovation resources on high-quality economic growth has become increasingly prominent. There are many researches on the influencing factors of innovation performance. The purpose of this study is to explore the factors that affect the innovation performance of high-end manufacturing clusters in China based on the dual perspectives of digital economy and innovation network. A total of 194 valid questionnaires were collected. And structural equation modeling has been used to test the proposed research models and hypotheses. The results indicated that, the higher the centrality of the innovation network, the more the cluster enterprises can play the centrality advantage, which has a significant positive impact on the innovation performance of the cluster. Similarly, both the strength and density of innovation networks also impacted on cluster innovation performance, but to a lesser extent. We also found that the digital empowerment derived from the digital economy can get rid of the limitations caused by spatial distance and lead to the improvement of resource utilization, which plays a positive moderating role between innovation network and innovation capacity. Implications for digital economy and innovation networks to improve the quality of innovation performance are provided.

## Introduction

With a new round of scientific and technological revolution and industrial revolution sweeping the world, promoting the development of digital economy has become the only way for China’s future development (Ma, 2019). The development and innovation of digital industry has become the core driving force of digital economy (Li et al., 2022). As the first driving force of social development and the strategic support of modern economic system, innovation drive plays an increasingly important strategic role in the development path of the new era (Xie et al., 2020; Hong, 2022). Under the new pattern of China’s future development, the innovation environment of emerging high-end industries has undergone tremendous changes, and the difficulty of innovation is also rapidly increasing (Chen and Yang, 2021). The complexity and subjectivity of technological innovation determines that enterprises cannot rely on limited internal resources to complete innovation activities, and need to use networked and cooperative organizational forms to form the maintenance capacity of innovation networks (Wang, 2022). At present, innovation network has become an important consideration for the innovation advantage of enterprises in emerging industry clusters. The innovation network derived by enterprises based on their own social capital is conducive to the flow, diffusion and integration of innovation resources (Zheng et al., 2020). It also helps to enhance the innovation level of enterprises in the cluster, break through the technological barriers of the industry, and provide support for their innovation performance (Xie et al., 2014). Therefore, an in-depth understanding and study of the innovation performance of cluster firms from an innovation network perspective has become the key to be urgently addressed.

The development of the digital economy can not only boost the intelligent development of the industrial chain (Ren and He, 2022), supply chain (Watanabe et al., 2018), and value chain (Szalavetz, 2020), but also effectively improve the economy, generate social benefits (Cui et al., 2020), and become the driving force for innovation and productivity growth. This is of great significance for improving regional innovation efficiency (Yuan and Gao, 2022) and high-quality development (Zhao et al., 2020). In the context of the digital economy, the derived digital empowerment can also effectively lead the innovation and development of the manufacturing industry. It stimulates advanced manufacturing enterprises to change their development models, break through the shackles of inherent traditional concepts, and actively carry out continuous innovation activities, effectively leveraging advanced manufacturing enterprises to achieve subversive innovation (Zheng, 2020). Digital empowerment can also provide serial nodes for data-driven innovation, prompting enterprises to use network platforms to continuously improve their technological innovation level and digital service application capabilities, thereby promoting regional innovation capabilities to continue to rise (Zhao, 2021).

Innovation is an exceptionally frequent and complex process. Under the general trend of open innovation, the independent innovation method is gradually replaced by cooperative innovation, and the external innovation ability, resources, knowledge and social relationship of enterprises are increasingly obvious in promoting the technological innovation of enterprises. Among them, innovation performance is the benefits or results obtained by firms through activities such as technological innovation, product innovation, and process improvement (Saunila, 2016). The innovation network is the vehicle for collaborative innovation activities. Companies rely on the innovation network they are in to maintain a high rate of innovation and gain a sustainable competitive advantage (Guan and Liu, 2016). The existing literature has mainly studied regional innovation networks (Lyu et al., 2019), technological innovation networks (Jiang et al., 2020), collaborative innovation networks (Xie et al., 2016), and cluster innovation networks (Zhong and Tang, 2018; Yue and Zhao, 2022). Among these characteristics, the network density and network structure of cluster innovation networks can effectively leverage the relationships of external subjects that have been or are being established to gain more information and knowledge, positively affecting cluster innovation performance (He et al., 2018). Relying on the innovation network of cluster enterprises, enterprises gradually shift from unilateral “closed door” to open collaborative innovation. With this approach to innovation, firms are able to draw on a wider range of innovation elements, which is conducive to seizing potential innovation opportunities. However, most of the existing research objects are traditional or more maturely developed industrial cluster enterprises, less research is conducted on emerging technology cluster enterprises in the early stage of development, and there are some gaps in the research objects.

In the research, many scholars have found that the relationship between innovation network and innovation performance is not a simple direct interaction. In different circumstances, its effects are heterogeneous and different, and other factors will also play a moderating or mediating role in the relationship between the two (Mulyana and Wasitowati, 2021). Moreover, innovation network multidimensional factors may also have an impact on innovation performance. Different locations occupied by firms and different characteristics of the network can affect the allocation and access to innovation resources (Zhang et al., 2022). The close cooperative relationship between the principals and the core location of the network may have a positive impact on the technological innovation performance. Firms that occupy a core position in an industry cluster tend to have an advantage, and network relationships allow this advantage to be effectively enhanced, which in turn has a positive effect on innovation performance (Lin et al., 2016). Some scholars have also studied the internal relationship between cluster enterprise innovation network and innovation through the knowledge diffusion simulation model, and found that core enterprises play an important positive role in cluster enterprise innovation. However, the effect of bad knowledge absorption among network members may have an impact on this positive effect (Eyvazzadeh, 2021). After combing through the literature, it can be found that existing relevant studies tend to focus on multiple clusters at the macro level and not much research has been done at the micro level.

To enrich the research in the field of innovation network and innovation performance, this study analyzes the multi-dimensional impact of the digital economy and cluster enterprise innovation network on innovation performance from the macro-environment and micro-levels. The influence path is verified by the corresponding scale designed and combined the structural equation model, which systematically reveals the impact of innovation network structure on the innovation performance of cluster enterprises. By addressing the following research questions:

Question 1: How do digital economy and cluster enterprise innovation network structure characteristics affect enterprise innovation performance?
Question 2: With the many factors influencing digital economy, innovation networks and innovation performance, how can the question items on the variable test scale be selected to improve the credibility of the results?
Question 3: Can the introduction of SEM further validate the hypothesis of the relationship between digital economy, innovation networks and innovation performance of cluster firms and clarify their impact paths?

To answer the above three problems, this study dissects the intrinsic relationship between the digital economy, cluster firm network structure and innovation performance based on social capital theory, resource dependency theory and collaborative innovation theory. Therefore, relative to previous literature, the research contributions of this paper focus on the following aspects: First, it enriches the research related to the innovation network of enterprises in emerging industry clusters. Most of the existing literature focuses on enterprises in more mature industrial clusters. This study selects high-end manufacturing, an emerging industry based on major technological breakthroughs and significant development needs, and dissects the current situation of its cluster enterprise innovation network, closely following its innovation status and future development goals. It provides a reference for the research related to the innovation network of emerging enterprises; Second, this paper broadens the breadth of research on the innovation performance of cluster firms based on innovation networks and combined with the actual situation of specific cluster firms. Currently, scholars are rich in innovation performance of cluster firms, but most of them adopt a purely empirical approach to study the innovation performance of cluster firms based on data from firms in different clusters. This study selects firms within a specific single cluster and systematically investigates the actual situation of these firms with the help of an anonymous questionnaire. It analyzes the paths of action affecting the innovation performance of cluster firms in terms of several characteristics of the innovation network, expecting to complement the findings of existing studies on the innovation performance of cluster firms; Third, it further expands the research perspective on the factors influencing innovation performance. Most of the existing studies focus on innovation networks, and few studies have been conducted from the dual perspective of digital economy and innovation networks. Based on the digital economy and innovation network, this study analyzes the factors influencing the innovation performance of cluster enterprises from two perspectives: macro environment and micro level. It is expected to expand the research horizon of innovation performance of cluster enterprises and also provide policy support for promoting innovation development of high-end manufacturing cluster enterprises.

The subsequent part of this paper is organized as follows: Part II constructs a theoretical model to analyze the factors influencing the innovation performance of cluster firms, from which research hypotheses can be derived for empirical testing; Part III describes the empirical study design and conducting data quality tests; Part IV and Part V perform structural equation analysis and further discussion; Part VI summarizes the paper’s findings and makes policy recommendations.

## Theoretical basis and research hypothesis

### The impact of cluster firm network centrality on innovation performance

In cluster firm innovation networks, network centrality reflects the firm’s position in the network, indicates the strength and breadth of the firm’s linkages with other members, and reflects the firm’s degree of access to and control over diverse resources. In this study, the accumulation of mutual support behaviors and standards between enterprises and external members is regarded as the social capital of enterprises, and the connection is formed by the altruistic concept of individuals and the interaction or transaction between individuals. This connection is based on the network and can obtain more benefits from the network, which also means obtaining more social capital., When a firm is at the core of the network, the more ties it has with other members of the network, the higher the centrality of the network. For China’s high-end manufacturing cluster in a more central position in the network, they have richer access to resource elements, and can reduce the resource disadvantage caused by information asymmetry. The smooth exchange of information is also conducive to enterprises’ mastery of diverse and heterogeneous information, enjoying a clear information advantage to facilitate knowledge learning and resource integration (Dou and Wang, 2011). As a result, companies in core positions tend to have more advantages in patented technologies, new product development, and sales. Moreover, diversified information not only increases the probability of obtaining information conducive to independent innovation but also provides more ideas for enterprise technological innovation and promotes the implementation of innovation-oriented strategies of enterprises (Wang and Wang, 2020). To sum up, we propose the following hypothesis.

*H1*: Cluster firms' innovation network centrality has a positive effect on innovation performance

### The impact of cluster firm linkage strength on innovation performance

Network strength refers to the strength of the connection between the subjects in the network and reflects the reliability of the relationship between the subjects of the innovation network. Based on resource dependency theory, the organization cannot own and control all resources, and differential competition due to different resources held by scarce resource organizations (Hessels and Terjesen, 2010). And the key resources that each business organization needs to maintain its core competitive advantage often cannot be generated on its own. This results in the development and actions of the company being influenced by external factors and the need to rely on interaction with the external environment to obtain the resources and information it needs (Wry et al., 2013). The strong connection of enterprise innovation network is conducive to the establishment and maintenance of good internal relationships, enhancing trust between enterprises, which in turn leads to cooperative relationships and promotes the sharing of innovation directions and theoretical knowledge. For China’s high-end manufacturing cluster in the rapid development period, only a small number of enterprises have more communication and cooperation with each other. However, as the number of cluster enterprises is still growing at this stage, there is still a large potential for exploiting the intrinsic relationship value. The communication and cooperation between cluster enterprises have become more in-depth, lasting and reliable due to the high trust in the strong connection network relationship (Dai and Liu, 2018). The higher the strength of relationships in the innovation network, the higher it helps to increase the speed of innovation resource transfer in the innovation network, the higher the frequency of resource replacement, knowledge transfer, and problem sharing and solving among enterprises, and the easier it is for cluster subjects to obtain useful information in the network and to absorb it into their innovation capabilities, improving the innovation performance of enterprises within the cluster. Hence, we propose:

*H2*: Cluster firms' innovation network strength has a positive effect on innovation performance

### The impact of the tightness of cluster subject ties to innovation performance

Network density refers to the closeness of the connection between enterprises and other organizations within the cluster, reflecting the degree of association and the degree of agglomeration of the innovation network. High network density means more high-frequency communication and in-depth cooperation among enterprises, which is conducive to the dissemination and diffusion of information and promotes the sharing and transfer of explicit and tacit knowledge (Ganguly et al., 2019), and also improves the integration of knowledge among subjects within the innovation network, increases the probability that individual enterprises can obtain effective information from it, and improves the innovation capability of enterprises (Xu et al., 2019). The increase in the frequency of communication and cooperation also tends to give rise to a standardized information and resource sharing mechanism, which in turn contributes to the construction of high trust relationships. Based on the theory of collaborative innovation, multiple subjects each provide their high-quality resources to achieve a common purpose, forming an associated contractual arrangement. At present, although there are industry chain collaborations and industry alliances composed of some enterprises in China’s high-end manufacturing cluster, the sharing of information and resources is limited to local, and most of the enterprises in China’s high-end manufacturing cluster have not yet achieved the construction of a high trust relationship. If there are more high-trust relationships, it helps companies to access knowledge resources and reduce the cost of relationship management and maintenance, which in turn boosts innovation performance (Wang and Liu, 2019). This is one of the important reasons that the current China’s high-end manufacturing cluster is implementing a whole industry chain layout and actively promoting industry-university-research cooperation. In addition, the close network relationship can also promote creative thinking and divergent thinking among the subjects of the innovation network, which can lead to more new creativity and ideas (Perkins, 2019), and energize the innovation of the cluster enterprises. As a result, the following hypothesis is made:

*H3*: Cluster firms' innovation network density has a positive effect on innovation performance

### The moderating role of digital economy between cluster innovation network and innovation performance

Due to the rapid development of the digital economy, digital technology is gradually embedded in various links such as production, research and development, and communication. Digital empowerment mainly relies on digital technology to enhance enterprise information transmission, data analysis, intelligent processing and other capabilities, and empowers the changes in the original production paradigm and business model of enterprises. China’s high-end manufacturing cluster innovation network is formed by the connection of multiple subjects. There is a certain spatial distance between each subject, and the communication between them depends to a certain extent on media communication. However, there is a certain time lag in traditional media communication. Digital technology can effectively overcome the limitations brought by time and space distance, make the connection between the innovation subjects in the cluster closer, and it is easier to promote the establishment of an innovation network. Digital empowerment urges enterprises to use digital technology to not only efficiently integrate information and resources in the network, but also to optimize resource allocation, which is conducive to the improvement of enterprise innovation efficiency (Yin and Tian, 2021). It can also reduce the cost of contact between various entities in the network, improve the efficiency of knowledge exchange, broaden the scope of communication, promote the development and deepening of collaborative innovation cooperation, and achieve the improvement of innovation efficiency (Hou and Gao, 2022). In addition, digital empowerment can promote the improvement of resource heterogeneity, provide more possibilities for reorganizing the combination of various elements with efficient intelligent processing capabilities, and make up for the lack of innovative product output caused by the lack of heterogeneity of existing network resources (Yang et al., 2021). In addition, the speed of information transmission under digital empowerment has increased by an order of magnitude (Liu et al., 2022). This makes all kinds of information in the innovation network more open and transparent. This can effectively reduce the information asymmetry between cluster enterprises, prevent some small and medium-sized enterprises from taking advantage of information to implement speculative behaviors, effectively reduce the “moral hazard” and other problems existing in the cooperative innovation of cluster enterprises, and promote China’s high-end manufacturing clusters to improve innovation performance.

In order to adapt to the research theme of this paper, we draw on the research perspectives of the above scholars to use digital empowerment to explain the role of the digital economy, and think that digital empowerment is the full application of digital technology by organizations to enhance or activate their information transmission, data analysis, intelligent processing and other capabilities. Digitally empowered China’s high-end manufacturing clusters have higher integration efficiency of innovative network resources and higher information transmission rates. This promotes diversified, in-depth, and high-quality collaborative innovation cooperation within the network, thereby improving innovation performance. Therefore, we propose the following three hypotheses:

*H4*: Digital empowerment has a positive moderating effect on cluster innovation network centrality and innovation performance

*H5*: Digital empowerment has a positive moderating effect on cluster innovation network strength and innovation performance

*H6*: Digital empowerment has a positive moderating effect on cluster innovation network density and innovation performance

**Figure 1:**
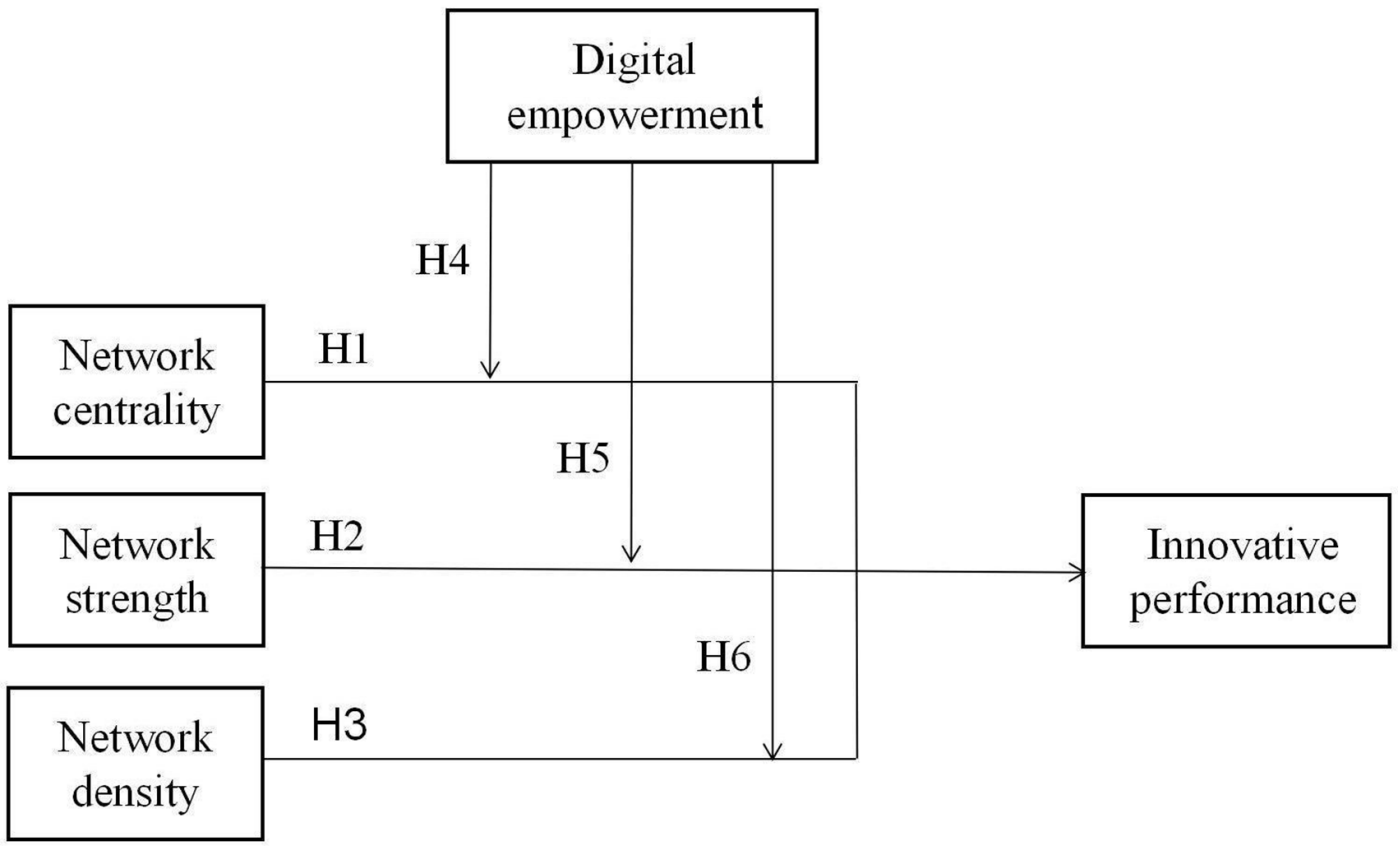
Theoretical framework of cluster innovation performance research.

The theoretical framework of cluster innovation performance research is illustrated in Figure 1.

## Research design and testing

### Selection of the study population

China’s high-end manufacturing is a high-end product of industrialization and digital development. It is a knowledge-intensive, technologically advanced, strategic industry with high product added value and good growth potential, and is the core of manufacturing development. While most traditional industries are capital-intensive and labor-intensive, highly substitutable, and susceptible to external fluctuations, most high-end manufacturing in China are knowledge-intensive and technology-intensive, and belong to industries with high growth potential and comprehensive benefits, producing products and providing services with high added value and high technology, which have a significant leading role in long-term economic and social development. This study takes into account the availability of data and the core element around which this paper revolves, innovation, and uses China’s high-end manufacturing, which is based on major technological breakthroughs and significant development needs, as a representative for empirical research.

### Research design options

Based on the research hypothesis and theoretical model, this paper selects cluster firm innovation network, digital economy and innovation performance as the main research variables, where the cluster firm innovation network variables include network centrality, network strength, and network density, and the research variables are measured by Likert 7-level scale to improve the accuracy and credibility of the research results.

#### Cluster enterprise innovation network

In this paper, the research on innovation networks of cluster firms is measured in three aspects: network centrality, network strength, and network density. Referring to the studies of Dou and Wang (2011), this paper designs four questions to measure the innovation network centrality of cluster firms. Drawing on Eisingerich et al. (2010) measures network strength in terms of direct linkage, information sharing, and resource sharing. Referring to the study of Yu et al. (2021), this paper designs a network density scale covering five measurement questions. As illustrated in Table 1.

**Table 1:** Cluster firm innovation network scale.

Dimension of the study variables	Title code	Title content
Network centrality	ZX1	Most companies in the cluster are aware of our technical capabilities and products
	ZX2	Other companies in the cluster can easily connect with us for technical communication
	ZX3	Other companies often exchange technology with cluster companies through us
	ZX4	When technical support is needed, cluster companies often want us to provide new knowledge or technology
Network strength	QD1	The enterprise is closely linked to other enterprises within the cluster
	QD2	Frequent sharing of resources between the enterprise and other enterprises within the cluster
	QD3	High exchange of information between the enterprise and other enterprises within the cluster
Network density	MD1	Closer communication and cooperation with upstream suppliers in the cluster than other companies
	MD2	Closer communication and cooperation with downstream suppliers in the cluster than other companies
	MD3	Closer communication and cooperation between our company and similar companies in the cluster than in other companies
	MD4	Closer communication and cooperation with upstream suppliers and research institutions in the cluster than other companies
	MD5	Closer communication and cooperation with non-research institutions in the cluster than with other companies

#### Digital empowerment meter

Combined with the previous definition of digital empowerment, digital empowerment refers to the full application of digital technology by organizations to enhance or activate their information transmission, data analysis, intelligent processing and other capabilities. This paper draws on previous research on digital empowerment and designs six items to measure. Digital empowerment (Lenka et al., 2017; Zhang et al., 2021). As illustrated in Table 2.

**Table 2:** Innovation performance scale.

Dimension of research variables	Title code	Title content
Digital empowerment	SJ1	The information technology introduced by the enterprise can enhance the management communication within the organization
	SJ2	Enterprises can continuously and real-time obtain various information inside and outside the enterprise
	SJ3	Information technology-related software and hardware facilities introduced by enterprises can enhance intelligent functions
	SJ4	Enterprises can quickly identify and properly allocate data resources between departments
	SJ5	Businesses are able to isolate valuable information from massive amounts of data
	SJ6	Enterprises can provide organizations with valuable predictive insights in design, R&D, production, marketing, finance and more based on massive data

#### Innovation performance scale

Due to the large number of fields covered within the China’s high-end manufacturing cluster and the uneven innovation level of enterprises within the cluster, the direct use of objective patent data may lead to bias in the research results. Therefore, this paper refers to the studies by Liu et al. (2016), and others to design the following six questions to measure innovation performance comprehensively. As illustrated in Table 3.

**Table 3:** Innovation performance scale.

**Dimension of research variables**	**Title code**	**Title content**
Innovative performance	CX1	High level of technical processes and procedures in the enterprise
	CX2	We have introduced more new production operations than our peers
	CX3	Higher ROI on our new products compared to our peers
	CX4	The higher success rate of technology conversion of enterprises compared to peers
	CX5	The company has a high proportion of new product sales to total sales compared to peers

### Questionnaire distribution and data collection

The questionnaire designed in this paper consists of the following three parts: (1) research-related instructions. The purpose of the questionnaire survey is clarified, and the requirements for filling out the questionnaire survey are made clear; (2) the basic situation of China’s high-end manufacturing enterprises. The basic situation covers the year of establishment, nature, size, and position of the interviewed China’s high-end manufacturing enterprises; (3) the core questions of the questionnaire. Based on the previously designed scale and using the seven-level Likert evaluation method, from 1 to 7 corresponded to completely un-conforming to completely conforming, respectively, and graded to measure the respondents’ recognition of the degree of conformity to the description of the question items. Finally, after setting up the preliminary questionnaire, a small sample of some respondents was tested to check the feasibility and credibility of the questionnaire.

The questionnaire was distributed in three main ways: (1) field visits. Reaching out directly to local high-end manufacturing enterprises, distributing and retrieving paper copies of questionnaires directly to middle and senior managers of enterprises, and briefly communicating on the situation related to innovation in high-end manufacturing clusters; (2) online network questionnaire. Because online management and communication are more frequent nowadays, enterprise managers and departments often connect online through WeChat, nail and other apps. Therefore, the use of QR codes and website links in the form of inviting business managers to fill out the questionnaire; (3) participate in the communication sessions held offline. Paper or electronic questionnaires were distributed to exhibitors at the venue or exhibition hall, and the form of filling out the questionnaires respected the wishes of the research subjects. Since a large number of high-end manufacturing enterprises participated in the event, and most of the participants were middle and senior management or technical R&D personnel, they were highly compatible with the research subjects of this paper. Referring to the suggestions of scholars, the sample size required for the measurement model should be more than five times that of the observed variable, and ten times is more appropriate. Too large a sample size can make the Chi-square value too sensitive (Bentler and Chou, 1987). Therefore, by October 30, 2021, 220 questionnaires were sent out for this survey, and 194 valid data were received after deleting the incomplete and unauthentic questionnaires, which met the needs of the sample.

Descriptive statistics of the 194 valid questionnaires formally distributed and collected reveal that most of the organizations in this study are 1–5 years old, small and medium-sized enterprises. This is because high-end manufacturing is a new industry, and many enterprises within the cluster were established relatively late and are still in the stage of rapid development. And most of the research subjects were located in Jiangxi, China, due to the impact of the COVID-19 pandemic. The participants of the questionnaire are basically middle and senior management and R&D personnel, who know the situation of collaborative innovation, innovation output and technology level of their companies. The anonymous completion of the questionnaire also guarantees the objective evaluation of the innovation situation of the enterprises to which they belong as much as possible.

### Data quality inspection

#### Harman’s single-factor test

The Harman’s single-factor test was used to test the data for common method deviation. The results were as follows: It showed that the Kaiser–Meyer–Olkin (KMO) values were 0.905, and the Bartlett sphericity test was conducted for Chi-square. The value was 2411.490, the df value was 253, and the significance level was *p* < 0.000. Therefore, the homologous deviation test can be performed using the Harman’s single-factor test. The results of the Harman’s single-factor test showed that five factors with characteristic roots greater than 1 were extracted. Of these, the variance explained by the first common factor is 36.616%, which is less than the generic critical value of 40%. Therefore, there was no serious homologous deviation in this study.

#### Reliability testing

To ensure the uniformity of the various indicators of the scale, based on the relevant studies in the existing literature, Cronbach’s alpha coefficient is chosen as the measure of reliability test in this paper, and the data are calculated by SPSS26.0 software, and the results are shown in Table 4. It can be found that the Cronbach’s alpha coefficient of network centrality variable is 0.845, the Cronbach’s alpha coefficient of network strength variable is 0.831, the Cronbach’s alpha coefficient of network density variable is 0.862, Cronbach’s alpha coefficient for digital empowerment is 0.896, and Cronbach’s alpha coefficient of innovation performance is 0.867. The Cronbach’s alpha coefficient for all five study variables is greater than 0.7, the CITC value for each question item is greater than 0.5, and the Cronbach’s alpha decreases to varying degrees when the items are removed. This indicates that the scale has a high reliability.

**Table 4:** Results of the reliability test.

Dimension of research variables	Title code	CITC	Cronbach’s Alpha after removal of terms	Cronbach’s alpha
Network centrality	ZX1	0.665	0.814	0.845
	ZX2	0.663	0.811	
	ZX3	0.721	0.786	
	ZX4	0.688	0.803	
Network strength	QD1	0.697	0.758	0.831
	QD2	0.680	0.775	
	QD3	0.691	0.764	
Network density	MD1	0.708	0.826	0.862
	MD2	0.606	0.852	
	MD3	0.720	0.823	
	MD4	0.725	0.822	
	MD5	0.647	0.842	
Digital empowerment	SJ1	0.694	0.881	0.896
	SJ2	0.805	0.864	
	SJ3	0.704	0.879	
	SJ4	0.714	0.878	
	SJ5	0.646	0.889	
	SJ6	0.752	0.872	
Innovative performance	CX1	0.692	0.838	0.867
	CX2	0.684	0.841	
	CX3	0.659	0.846	
	CX4	0.706	0.835	
	CX5	0.706	0.835	

#### Validity testing

In this paper, we comb through existing studies, select mature scales with strong relevance, high reliability and validity to the research topic, combine them with the research object of the China’s high-end manufacturing cluster, and adjust the details of the scale to form a preliminary scale and test its convergent validity with AVE value, combined reliability and other indicators to better verify the authenticity and applicability of the data.

If the measured combined reliability (CR) of the scale is higher than 0.7 and the average variance extracted (AVE) value is more than 0.5, it means that it has good convergent validity. In this paper, the convergent validity of the formal scale was further tested using SPSS 26.0 software. The results of the convergent validity test in Table 5 show that the combined reliability of the five variables is above 0.8, which is greater than 0.7; the average variance extracted (AVE) is also greater than 0.5, which indicates that the formal scale has good convergent validity.

**Table 5:** Convergent validity test results.

Dimension of the study variables	Title code	Standardization factor	Combined reliability	AVE
Network centrality	ZX1	0.716	0.848	0.582
	ZX2	0.749		
	ZX3	0.822		
	ZX4	0.761		
Network strength	QD1	0.793	0.831	0.622
	QD2	0.789		
	QD3	0.783		
Network density	MD1	0.774	0.864	0.561
	MD2	0.666		
	MD3	0.789		
	MD4	0.801		
	MD5	0.704		
Digital empowerment	SJ1	0.741	0.897	0.593
	SJ2	0.860		
	SJ3	0.740		
	SJ4	0.777		
	SJ5	0.693		
	SJ6	0.800		
Innovative performance	CX1	0.775	0.897	0.593
	CX2	0.725		
	CX3	0.738		
	CX4	0.746		
	CX5	0.775		

#### Relevance analysis

Based on the data collected from the enterprises within China’s high-end manufacturing cluster, correlation analysis was conducted to test the degree of relationship between several variables. When the correlation coefficient is higher than 0.7, it indicates that there may be a co-linearity problem in this sample; while when the probability p of the correlation coefficient is greater than 0.05, it indicates that there is no significant correlation between the two variables.

The results of the correlation coefficients of the main variables of this study are presented in Table 6 and the correlation coefficients of the observed variables of the independent and dependent variables are presented in Table 7. It can be found that the correlation of the observed variables of each variable is basically significant at the two-tailed significance level of 0.01, the correlation of observed variables is basically significant, and there is also significant correlation between network centrality, network strength, network density, digital empowerment and innovation performance, and there is no collinearity problem.

**Table 6:** Correlation analysis of study variables.

	Network centrality	Network strength	Network density	Digital empowerment	Innovative performance
Network centrality	1				
Network strength	0.456^**^	1			
Network density	0.513^***^	0.473^***^	1		
Digital empowerment	0.323^***^	0.192^***^	0.323^***^	1	
Innovative performance	0.581^***^	0.514^***^	0.545^***^	0.371^***^	1

***p < 0.001, **p < 0.01.

**Table 7:** Correlation analysis of observation variables.

	CX1	CX2	CX3	CX4	CX5
ZX1	0.404^***^	0.259^***^	0.373^***^	0.241^***^	0.390^***^
ZX2	0.496^***^	0.388^***^	0.410^***^	0.344^***^	0.451^***^
ZX3	0.467^***^	0.327^***^	0.501^***^	0.338^***^	0.491^***^
ZX4	0.443^***^	0.327^***^	0.405^***^	0.370^***^	0.381^***^
QD1	0.409^***^	0.331^***^	0.306^***^	0.348^***^	0.322^***^
QD2	0.367^***^	0.346^***^	0.443^***^	0.348^***^	0.352^***^
QD3	0.341^***^	0.396^***^	0.339^***^	0.386^***^	0.350^***^
MD1	0.407^***^	0.288^***^	0.366^***^	0.320^***^	0.332^***^
MD2	0.374^***^	0.283^***^	0.338^***^	0.274^***^	0.337^***^
MD3	0.432^***^	0.269^***^	0.347^***^	0.344^***^	0.350^***^
MD4	0.453^***^	0.305^***^	0.441^***^	0.330^***^	0.355^***^
MD5	0.440^***^	0.316^***^	0.315^***^	0.425^***^	0.397^***^
SJ1	0.242^***^	0.256^***^	0.353^***^	0.188^***^	0.329^***^
SJ2	0.185^***^	0.214^***^	0.264^***^	0.172^**^	0.273^***^
SJ3	0.206^***^	0.212^***^	0.279^***^	0.175^**^	0.281^***^
SJ4	0.234^***^	0.202^***^	0.233^***^	0.166^**^	0.243^***^
SJ5	0.280^***^	0.331^***^	0.240^***^	0.186^***^	0.275^***^
SJ6	0.256^***^	0.247^***^	0.293^***^	0.209^***^	0.306^***^

***p < 0.001.

## Structural equation analysis

The structural equation model generally includes a measurement model and a structural model, and the measurement model is mainly used to analyze the relationship between latent and observed variables, and the classical measurement model equation is shown below:


(1) X=Λxξ+δ.

(2) Y=Λyη+ε.

Where *X* is a vector of exogenous observed variables and *Y* is a vector of endogenous observed variables; ^*x* and ^*y* are indicator variables, *δ* and *ε* are measurement errors of exogenous and endogenous observed variables; *ξ* is an exogenous latent variable, and *η* is an endogenous latent variable.

Structural models are then used to analyze the interactions between variables, and the classical structural model equation is shown below:


(3) η=Bη+Γξ+ξ.

Where B is the structural coefficient matrix of the relationship between the endogenous latent variables; Г is the structural coefficient matrix of the relationship between the endogenous latent variables and the exogenous latent variables, and *ζ* is the disturbance factor or residual value in the structural model.

### Structural equation modeling

**Figure 2:**
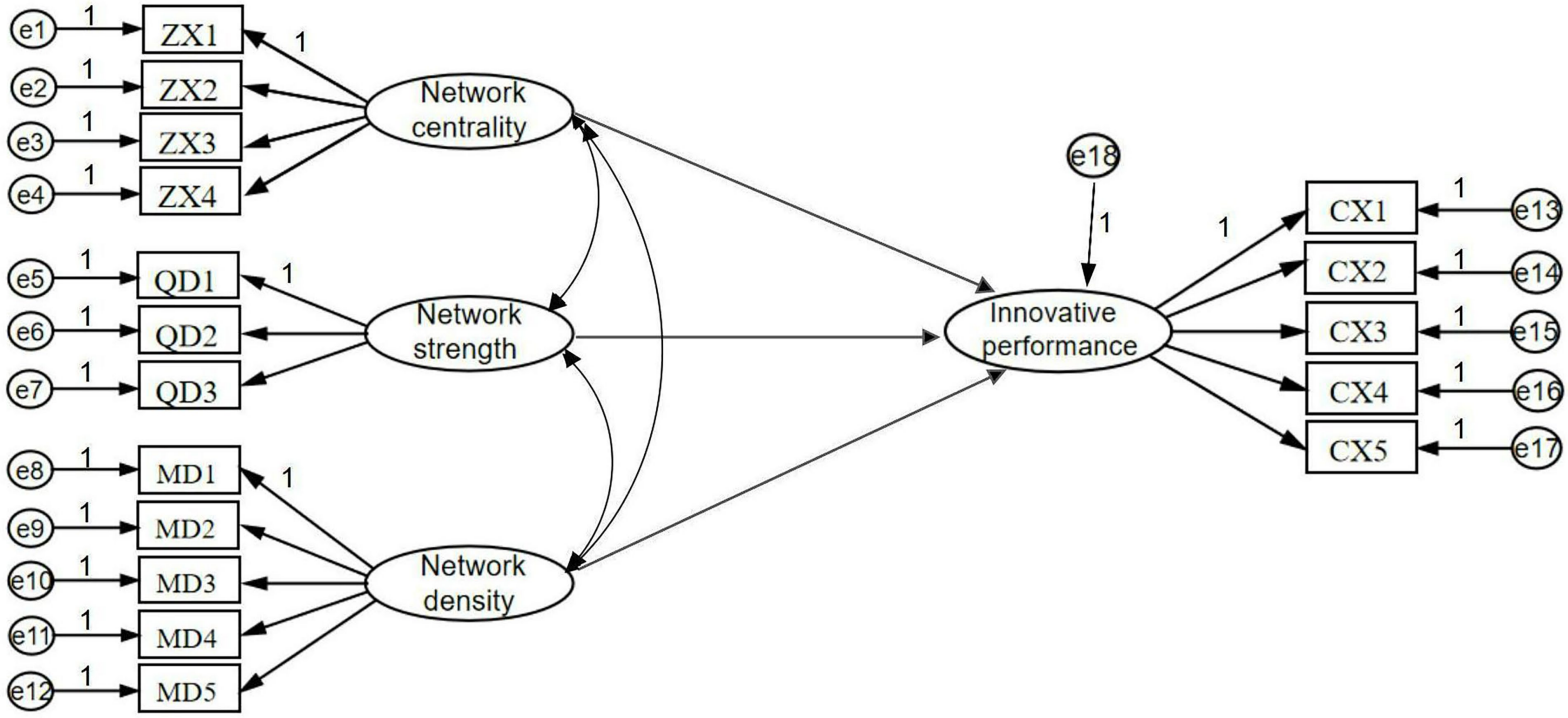
Structural equation model diagram.

Based on the validated factor analysis test of the measurement model passing, this section uses AMOS 26.0 software to construct the structural equation model shown in Figure 2, which includes four latent variables (network centrality, network strength, network density, and innovation performance) and 17 observed variables.

### Fit test

**Figure 3:**
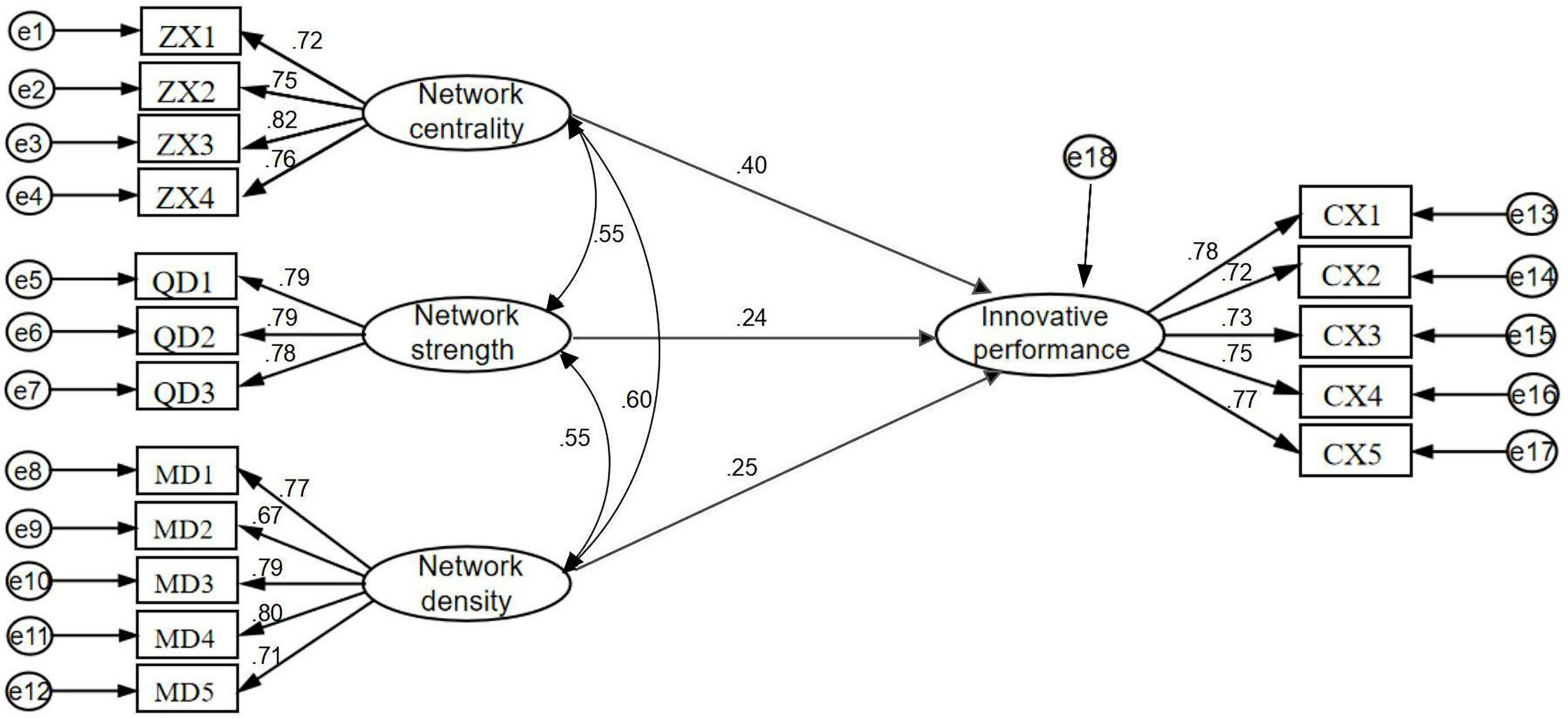
Structural equation path results.

Since the validated factor measures of an innovation network and innovation performance performed well with the convergent validity test results, this subsection is based on the three research hypotheses proposed above on innovation network and innovation performance of cluster firms, and after matching the corresponding data, it is obtained as in Figure 3.

From the output in Table 8, we can find that the *X*^2^/*df* value of the model is 1.177, which is close to 1, and the fitness is good; the GFI is 0.924, TLI value is 0.985, CFI value is 0.988, and IFI measurement is 0.988, the values of the selected indicators are higher than 0.9, and the RMSEA and SRMR values do not reach 0.05, which meet the fitness requirements.

**Table 8:** Structural equation model fitness.

Fitted indicators	Fit results	Adaptation decision
X^2^/df	1.177	Yes
GFI	0.924	Yes
IFI	0.988	Yes
CFI	0.988	Yes
TLI	0.985	Yes
RMSEA	0.030	Yes
SRMR	0.041	Yes

### Path analysis of the impact of cluster firm innovation networks on innovation performance

The results of the fit test of the structural equations show that the constructed structural equation model fitness indicators all meet the validation requirements. Therefore, this subsection continues to use the path coefficients calculated by AMOS 26.0 for each dimension of the innovation network of cluster firms on innovation performance (referring to Table 9) to test the hypothesis proposed in this paper regarding the relationship between the innovation network of cluster firms and innovation performance. When the *p* value is less than 0.05, it means that the path is significant; when the standardized path coefficient corresponding to the path relationship is positive, it means that the relationship between the two is positively influenced. From the results of the path coefficients of the structural equations in Table 9, it can be seen that the standardized path coefficient value of network centrality on innovation performance is 0.404 with a significance of 0.000, which is less than 0.05, implying that the network centrality of the innovation network of cluster firms significantly and positively affects innovation performance and hypothesis H1 holds. The standardized path coefficient value of innovation network strength on innovation performance is 0.240 with a significance of 0.006, which is less than 0.05, implying that network strength positively affects innovation performance and is significant at the 5% level and hypothesis H2 holds. The standardized path coefficient value of innovation network density on innovation performance is 0.253 with a significance of 0.005, which is less than 0.05, implying that network density has a significant positive effect on innovation performance and hypothesis H3 holds. Therefore, all three dimensions of an innovation network can significantly and positively affect innovation performance, that is, the higher the network centrality, strength, and density of the enterprise innovation network, the more conducive to the transfer of information resources of each enterprise in the cluster, higher resource convergence and allocation efficiency are also more capable of enhancing the innovation capacity of enterprises, promoting technological innovation into output and improving the innovation performance of enterprises.

**Table 9:** Impact path coefficients.

Path relation			Standardized path coefficient	Non-standardized path coefficient	Standard error	*T* value	*p* value	Assumption	Hypothesis testing
Innovative performance	←	Network centrality	0.404	0.363	0.086	4.205	^***^	H1	Establish
Innovative performance	←	Network strength	0.240	0.238	0.087	2.748	0.006	H2	Establish
Innovative performance	←	Network density	0.253	0.255	0.091	2.812	0.005	H3	Establish

***p < 0.001.

By comparing the standardized path coefficient values, it can be found that the path coefficient of network centrality reaches 0.404, which is about 0.15 higher than network intensity and network density, indicating that network centrality can have a stronger positive impact on innovation performance. This may be because high-intensity and high-density innovation networks have a more macroscopic contribution to innovation performance, and that network centrality enables firms within a cluster to access innovation resources and information more quickly and directly. A strongly connected and tight network is conducive to building connections among cluster firms, helping non-core firms in the cluster to break through resource and information barriers and providing a boost to their innovation development. However, it is worth noting that the enterprises originally in the center can also enjoy the “benefits” of high-intensity and high-density innovation network.

## The moderating effect test of digital empowerment

In order to test the moderating effect of digital empowerment between cluster innovation network and innovation performance, and to further analyze the impact of China’s high-end manufacturing cluster innovation network on innovation performance under different digital empowerment scenarios, this paper refers to existing research and incorporates digital empowerment into research model. We have taken the cluster innovation network as the independent variable, digital empowerment as the moderator variable, and innovation performance as the dependent variable for standardization, used SPSS 26.0 software to conduct hierarchical regression analysis to test the moderating effect. First, bring the independent variable and dependent variable into the regression equation (Model 1). Secondly, the digital enabling interaction term is brought into the regression equation (Model 2). The moderating effect analysis of digital empowerment is shown in Tables 10–12. Among them, in Model 1, the centrality, strength and density of the innovation network are used as independent variables, and the R12 obtained after calculation are 0.375, 0.342, and 0.340, respectively. In Model 2, the interaction term is used to bring into the model, and the calculated R22 are 0.425, 0.368, and 0.364, respectively, and the *R*^2^ coefficients are all positive and significantly improved. The results show that the positive moderating effect of digital empowerment exists, and hypotheses H4, H5, and H6 are all established.

**Table 10:** Test results of the moderating effect of digital empowerment between cluster innovation network centrality and innovation performance.

Variable	Innovative performance					
	Model 1			Model 2		
	Standardized coefficient	S.E.	*t*	Standardized coefficient	S.E.	*t*
Network centrality	0.515	0.053	8.519^***^	0.567	0.052	9.525^***^
Digital empowerment	0.205	0.051	3.398^**^	0.312	0.054	4.887^***^
Network centrality × digital empowerment				0.259	0.029	4.046^***^
*R* ^2^	0.375			0.425		
Adjusted *R*^2^	0.369			0.416		
Δ*R*^2^	0.375			0.050		
*F*	57.387^***^			16.372^***^		

***p < 0.001, **p < 0.01.

**Table 11:** Test results of the moderating effect of digital empowerment between cluster innovation network strength and innovation performance.

Variable	Innovative performance					
	Model 1			Model 2		
	Standardized coefficient	S.E.	*t*	Standardized coefficient	S.E.	*t*
Network strength	0.460	0.054	7.686^***^	0.440	0.053	7.433^***^
Digital empowerment	0.283	0.050	4.731^***^	0.323	0.051	5.339^***^
Network strength x Digital empowerment				0.167	0.039	2.813^**^
*R* ^2^	0.342			0.368		
Adjusted *R*^2^	0.335			0.358		
Δ*R*^2^	0.342			0.026		
*F*	49.557^***^			7.915^**^		

***p < 0.001, **p < 0.01.

**Table 12:** Test results of the moderating effect of digital empowerment between cluster innovation network density and innovation performance.

Variable	Innovative performance					
	Model 1			Model 2		
	Standardized coefficient	S.E.	*t*	Standardized coefficient	S.E.	*t*
Network density	0.475	0.059	7.648^***^	0.514	0.060	8.184^***^
Digital empowerment	0.218	0.052	3.508^**^	0.275	0.054	4.253^**^
Network density × digital empowerment				0.175	0.032	2.699^**^
*R* ^2^	0.340			0.364		
Adjusted *R*^2^	0.333			0.354		
Δ*R*^2^	0.340			0.024		
*F*	49.212^***^			7.287^**^		

***p < 0.001, **p < 0.01.

**Figure 4:**
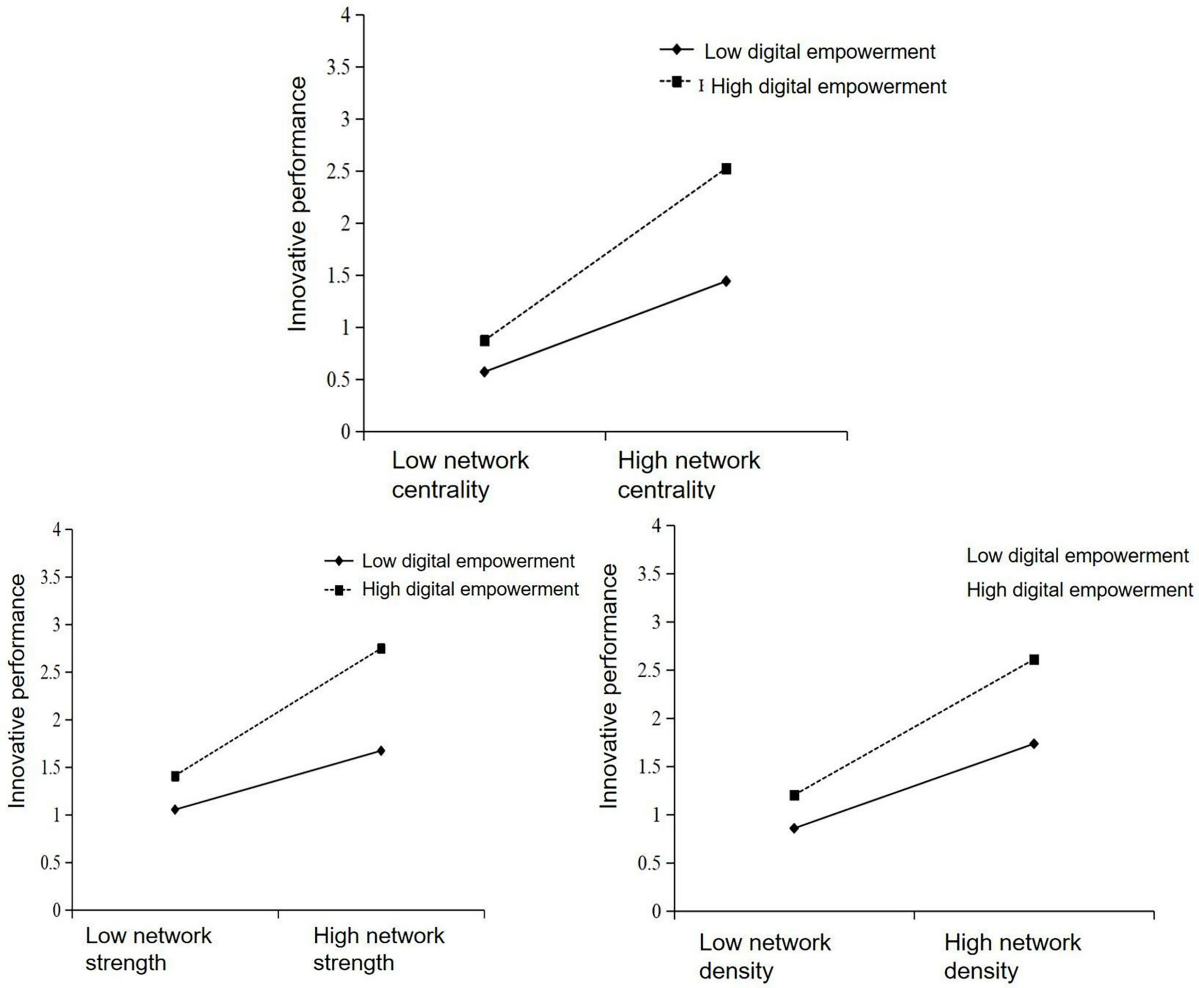
Digital empowerment regulating innovation network and innovation performance.

This study draws the relationship between innovation network centrality, network strength, network density and innovation performance under different grouping situations of digital empowerment, as shown in Figure 4. The research results clearly demonstrate and distinguish the difference in the slope of the relationship between cluster innovation networks and innovation performance under high and low levels of digital empowerment, to reveal the moderating role of digital empowerment between cluster innovation networks and innovation performance.

It can be seen that in the case of low digital empowerment, the slope of the positive impact of cluster innovation network centrality, network strength and network density on innovation performance is smaller than that in the case of high digital empowerment, which indicates that high digital empowerment level can enhance the impact of innovation network centrality, network strength and network density on innovation performance, and further verifies H4, H5 and H6.

## Discussion and conclusions

The results of this study improve our understanding of the relationship between the digital economy, cluster firm innovation networks, and innovation performance.

Regarding the relationship between innovation networks and innovation performance, based on the three dimensions of structural embeddedness proposed by Zukin and DiMaggio (1990), existing research revolves around the relationship between network structural characteristics and innovation performance:

1. Innovation network strength and innovation performance. One view is that weak linkage as an information bridge can avoid the repetition of similar information brought by strong linkage, and its transmission of heterogeneous information is more conducive to enterprise technology innovation (Matthew, 1998; Wang et al., 2021). Another view, which shares the results of this study, is that tacit knowledge transfer and high levels of trust resulting from strong ties will strongly contribute to firm technological innovation (Xie et al., 2014). Strong linkages are a good incentive for companies to exchange resources and information with other entities at a lower cost, resulting in deeper understanding, higher network cohesion, and similar corporate culture and values (Zhen et al., 2020). At the same time, strong network ties can reduce the uncertainty of the innovation environment, make the cooperation among network members more stable, help the formation of network norms and guidelines, and provide continuous knowledge interaction (Ren, 2010; Ren et al., 2011);
2. Innovation network density and innovation performance. The results of this study show that innovation network density is positively related to innovation performance. Cluster firms can effectively use the relationships of external subjects that have been or are being established to gain more information and knowledge through innovation network resources, and high-density innovation networks make innovation activities more meaningful (Eisingerich et al., 2010; Mors, 2010; He et al., 2018). However, some scholars believe that occupying a strong core position will make enterprises too embedded in a certain R&D network, which is not conducive to the production of new knowledge and new technologies (Zhang et al., 2017). Currently, it is still the dominant view among scholars that a high-density innovation network is more conducive to innovation performance. The higher the network density, the more connections and interactions between firms, the more efficient the knowledge flow and the higher the innovation performance of firms (Phelps, 2010). The high-density network is conducive to building connections among the enterprises in the cluster, helping non-core enterprises in the cluster to break through resource and information barriers, and providing a boost to their innovation and development;
3. Innovation network centrality and innovation performance. In line with the results of the vast majority of studies (Wang and Liu, 2019), this study shows that the higher the centrality of the innovation network of clustered firms, the more beneficial the firm’s innovation performance. Companies at the center of the network will occupy the dominant position in the network and have priority over other companies in terms of the breadth and depth of access to heterogeneous resources. Moreover, enterprises with higher centrality control the flow of information resources in the network, which improves the status of enterprises in the network and facilitates the timely and more favorable transformation of resources, thus improving the innovation performance of enterprises (Ibarra and Andrews, 1993; Du and Liu, 2021).
  Therefore, the conclusions of this study are as follows. The network centrality, network strength, and network density of the innovation network of cluster firms can positively influence the innovation performance of firms in the cluster. Among them, the centrality of innovation network of cluster enterprises can have a direct impact on the exchange of resources and information within the network. The higher centrality can stimulate the efficient allocation of resources within the cluster and increase the exchange and utilization of knowledge and technology by enterprises. It significantly and positively affects the innovation performance of cluster firms; The strength of the innovation network determines the depth and frequency of node interaction activities in the network. The higher the strength, the higher the trust of the innovation network and the higher the willingness to collaborate and innovate. Therefore, it is more inclined to release resources, information and other innovation factors into the network, and each cluster enterprise can obtain more advantageous resources from it, which in turn improves the enterprise innovation performance; In a high-density innovation network, nodes tend to be more closely connected to each other and innovation boundaries are relatively blurred. This makes inter-organizational collaborative innovation less likely to be hindered by communication barriers, which can promote the diversification of innovation activities and positively influence the innovation performance of cluster firms. Therefore, the innovation network of enterprises in high-end manufacturing clusters is not only the need to strive toward a high-density and high-intensity network, but also to enhance its network centrality. They should strive to occupy the central position and carry out cooperative exchanges with more innovation network subjects. In turn, they will help improve the innovation performance of the enterprises themselves.

At the same time, digital empowerment in the context of the digital economy is becoming more and more important. Through the moderating effect test, it is found that digital empowerment has a positive moderating effect in the cluster innovation network and innovation performance. It means that the cluster makes full use of digital technology, which can improve the innovation network centrality, network strength, and network density to a certain extent to promote the innovation performance of the cluster. This is largely consistent with the findings of previous scholars (Hou and Gao, 2022). Digital technology in the era of digital economy can enable efficient integration of information and resources within the network, which helps to achieve the goal of optimal resource allocation and achieve the improvement of enterprise innovation efficiency (Li et al., 2020). With digital empowerment, various types of information in innovation networks are more open and transparent, which contributes to the positive effect of innovation networks on innovation performance.

In summary, in order to effectively improve the innovation performance of cluster enterprises, it is necessary to further improve the innovation network of cluster enterprises. And the cluster network needs to be built with the help of digital technology. In this way, the strength of the connection between network nodes is consolidated, the density of the network is enhanced, and the centrality of the network needs to be improved in particular.

## Suggestions

As far as the enterprise level is concerned, on the one hand, enterprises should improve the cluster enterprise innovation network and strengthen enterprise collaborative innovation. The improvement of innovation performance of cluster enterprises is inseparable from the innovation network. In order to avoid being trapped in “information silos,” enterprises should make full use of their own social capital and extend their existing innovation networks. They should actively build relationships with universities, research institutions, government, and financial institutions. They can start innovative collaborations with companies of different sizes and break down information barriers between companies; They can form a good school-enterprise partnership with universities and research institutions to achieve breakthroughs in key technologies; They can also maintain high-intensity networking relationships with financial institutions to finance breakthrough innovation activities for companies. In short, the enterprises need to enrich the types of nodes in the innovation network of cluster enterprises, so that they can realize organic integration with other organizations through the network. At the same time they should enhance the frequency and quality of technology exchange, so that the innovation activities and innovation output of high-end manufacturing cluster enterprises can achieve a change from quantity to quality.

On the other hand, enterprises need to integrate digital technology, deepen their innovation network exchange mechanisms and optimize their internal governance. China’s high-end manufacturing cluster has been developed for a relatively short period, and the innovation network built as a whole stay on the surface of basic business cooperation, which is not conducive to the formation of a benign competitive atmosphere in the cluster in the long run. Therefore, in order to improve the existing communication mechanism, modern data transmission, analysis and processing capabilities can be used by means of digital technology to make the communication efficiency between the upper, middle and lower reaches of the industrial chain more efficient. The high network strength also enhances the sharing frequency of knowledge and resources among enterprises in the industrial chain. An efficient communication mechanism can break through the information barrier in the innovation network, help each network entity match the innovation elements suitable for their own development needs in the innovation network, strengthen the communication and cooperation within the capital network, and stabilize the capital flow within the cluster. At the same time, we should attach importance to and improve the management ability of digital knowledge, information, technology and other innovation elements within the enterprise, so as to encourage the enterprise to give full play to digital technology means, realize the maximum utilization of limited innovation elements, and then transform them into their own innovation power to promote the overall innovation of the cluster.

From the government level, on the one hand, the government needs to enhance top-level design and expand the clustering effect of innovation networks. Compared with the traditional manufacturing industry, the innovation of high-end manufacturing enterprises often requires more capital cost and human cost. And high-end manufacturing industry is a new industry, the development model is difficult to completely replicate the development model of other industries. Therefore, the government, as a cluster manager, should enhance the top-level design in order to expand the clustering effect of cluster innovation network and boost the high-quality development of cluster innovation. The government can strengthen the cultivation and introduction of talents and boost the cooperation between industry, academia and research. It is possible to form a complex innovation network model of cross-regional, open “learning-enterprise” cooperation and innovation with the help of the digital technology research results of top scientific research institutions. In this way, it will promote the diversification of industry-university-research cooperation, promote the transformation of industry-university-research research achievements, and improve the innovation efficiency of cluster enterprises. It can also deepen the local brand effect of high-end manufacturing industry and accelerate the localization of introduced leading projects. In this way, we can absorb innovation elements and bring into play the clustering effect of innovation network.

On the other hand, it is possible to strengthen the construction of digital infrastructure, establish a multi-dimensional guarantee mechanism, and optimize the enterprise innovation environment. Vigorously promote the development of the digital economy, pay attention to the construction of digital infrastructure. And actively cultivate new kinetic energy for digital development and play the potential role of digital empowerment, thereby promoting the output and agglomeration of innovative elements of regional cluster enterprises. At the same time, financial support shall be given to enterprises, such as setting up special subsidy funds for small-scale enterprises, providing technical support, implementing incentive policies by means of dynamic innovation performance evaluation, or giving credit preference to small and medium-sized enterprises with difficulties in development, and rewarding enterprises with initial success in innovation, so as to stabilize the cash flow and capital flow of enterprises. In addition to relying on the government’s own funds to support the enterprises in the cluster, the government can also be used as a medium to build a financing system for the enterprises in the industrial cluster, improve the capital turnover rate of the enterprises in the cluster, shorten the R & D cycle, fully mobilize the enthusiasm of enterprises in scientific research, and improve the overall innovation output.

## Implications for research and practices

### Theoretical implications

Firstly, the study enriches the research on innovation networks of emerging industry cluster enterprises. In the past, scholars’ research on the innovation network of cluster enterprises mainly focused on the more maturely developed industry cluster enterprises, and there was less research on emerging industries with shorter establishment times. This study combines the actual situation of China’s high-end manufacturing cluster enterprises, analyzes the current situation of the innovation network of emerging industry cluster enterprises, closely follows the current situation and future development goals of cluster enterprises, and proposes the sustainable development model for the innovation network of cluster enterprises, which provides a reference for the innovation networks of emerging enterprises.

Secondly, based on innovation networks, this paper broadens the breadth of research on the innovation performance of cluster firms by incorporating the actual situation of specific cluster firms. Currently, scholars are richer in innovation performance of cluster firms. However, most of them adopt a purely empirical approach, based on data from firms in different clusters. This study selects firms within a single cluster and analyzes the paths of action affecting the innovation performance of cluster firms in terms of multiple characteristics of the innovation network, with a view to complementing the findings of existing studies on the innovation performance of cluster firms.

### Implication for practices

Firstly, this study encourages cluster enterprises to actively use their own innovation networks. The construction of domestic industrial clusters has achieved initial results, but the independent innovation of some cluster enterprises is weak and the sustainability of innovation is low. From the perspective of digital economy and cluster enterprise innovation network, this study discusses how to improve the innovation performance of industrial cluster enterprises, create industrial clusters with core competitiveness, and achieve high-quality development of clusters. By optimizing the network, the cluster will promote the integration of internal heterogeneous resources and technical knowledge, improve the overall innovation ability of cluster enterprises, and further help improve the innovation performance of enterprises around the core vision of the cluster.

Secondly, this study provides support for the formulation of policies to promote the innovation and development of high-end manufacturing cluster enterprises. For cluster managers and enterprise subjects, an efficient and stable enterprise partnerships can reconstruct new industrial ecology, accelerate the iterative renewal of innovation technologies, and expand the growth space of internal enterprises. This study analyzes the influence of the network centrality, network strength, and network density of the innovation network of China’s high-end manufacturing cluster enterprises on innovation performance, which provides some theoretical basis for relevant government departments to clearly and accurately understand the innovation development of enterprises, allows the government to understand the development potential and development dilemma of enterprises within the industry cluster, and provides decision support for the innovation development of cluster enterprises based on the research findings.

## Limitations and future research

This paper analyzes the impact of network centrality, network strength, network density, and digital empowerment on the innovation performance of the innovation network of China’s high-end manufacturing cluster enterprises based on an in-depth study of the enterprises within China’s high-end manufacturing cluster. Although some valuable suggestions are proposed for the innovation development of the cluster enterprises, there are some limitations in this paper due to the constraints of resource availability, research subjects, time, and other subjective and objective conditions, which can be further improved and deepened in future research.

Firstly, China’s high-end manufacturing cluster enterprises are widely distributed, with a large number of small and medium-sized enterprises. So the information that can be collected is relatively limited. Most of the information used in this paper comes from the subjective questionnaires and interviews of China’s high-end manufacturing cluster enterprises, except for some quantitative data provided by government departments during internet information retrieval and interviews, but the data obtained utilizing field research also guarantee the authenticity and reliability of the data results. As the development of cluster enterprises grows, more quantitative data can be used in subsequent studies to make the case study more intuitive.

Secondly, Since China’s high-end manufacturing cluster is still in the development period, based on the collation of available information and interviews, it is found that the main body of the current innovation network of the cluster enterprises is China’s high-end manufacturing enterprises, so the scale design of this paper is biased toward China’s high-end manufacturing enterprises themselves. However, the scale design also covers the innovation situation of enterprises and the cooperation and communication with other subjects, to be as close as possible to the actual situation of the case and the research topic. Due to the influence of COVID-19, the samples obtained in this paper are located in a relatively limited area, which may have an impact on the use and testing of the subsequent structural equation model. Therefore, in future studies, the collection of sampling information can be appropriately expanded to minimize the error of model testing and enhance the completeness of the study.

## Data availability statement

The raw data supporting the conclusions of this article will be made available by the authors, without undue reservation.

## Ethical statement

Following local legislation and institutional requirements, ethical review, and approval are not required for the current study. The participants provided their written informed consent to participate in this study. Relevant data were anonymously collected from the respondents based on the principle of voluntary participation.

## Author contributions

LZ proposes the research topic, obtains research funds, reviews and revises the research project. KX researches and organizes the literature, writes the preliminary draft of the paper, validates and verifies the experimental design. XG and YY conducts the questionnaire, collects and organizes the data, and visualizes the experimental results. All authors contributed to the article and approved the submitted version.

## Funding

The research was funded by the National Natural Science Foundation of China (Project no. 72262014); Jiangxi Social Science Planning Project (Project no. 21GL15); Jiangxi University Humanities and Social Sciences Project (Project no. GL21114).

## Conflict of interest

The authors declare that the research was conducted in the absence of any commercial or financial relationships that could be construed as a potential conflict of interest.

## Publisher’s note

All claims expressed in this article are solely those of the authors and do not necessarily represent those of their affiliated organizations, or those of the publisher, the editors and the reviewers. Any product that may be evaluated in this article, or claim that may be made by its manufacturer, is not guaranteed or endorsed by the publisher.
